# Loss of Dependence on Continued Expression of the Human Papillomavirus 16 E7 Oncogene in Cervical Cancers and Precancerous Lesions Arising in Fanconi Anemia Pathway-Deficient Mice

**DOI:** 10.1128/mBio.00628-16

**Published:** 2016-05-17

**Authors:** Soyeong Park, Jung Wook Park, Henry C. Pitot, Paul F. Lambert

**Affiliations:** Department of Oncology, McArdle Laboratory for Cancer Research, University of Wisconsin School of Medicine and Public Health, Madison, Wisconsin, USA

## Abstract

Fanconi anemia (FA) is a rare genetic disorder caused by defects in DNA damage repair. FA patients often develop squamous cell carcinoma (SCC) at sites where high-risk human papillomaviruses (HPVs) are known to cause cancer, including the cervix. However, SCCs found in human FA patients are often HPV negative, even though the majority of female FA patients with anogenital cancers had preexisting HPV-positive dysplasia. We hypothesize that HPVs contribute to the development of SCCs in FA patients but that the continued expression of HPV oncogenes is not required for the maintenance of the cancer state because FA deficiency leads to an accumulation of mutations in cellular genes that render the cancer no longer dependent upon viral oncogenes. We tested this hypothesis, making use of *Bi-L E7* transgenic mice in which we temporally controlled expression of HPV16 E7, the dominant viral oncogene in HPV-associated cancers. As seen before, the persistence of cervical neoplastic disease was highly dependent upon the continued expression of HPV16 E7 in FA-sufficient mice. However, in mice with FA deficiency, cervical cancers persisted in a large fraction of the mice after HPV16 E7 expression was turned off, indicating that these cancers had escaped from their dependency on E7. Furthermore, the severity of precancerous lesions also failed to be reduced significantly in the mice with FA deficiency upon turning off expression of E7. These findings confirm our hypothesis and may explain the fact that, while FA patients have a high frequency of infections by HPVs and HPV-induced precancerous lesions, the cancers are frequently HPV negative.

**Importance  ** Fanconi anemia (FA) patients are at high risk for developing squamous cell carcinoma (SCC) at sites where high-risk human papillomaviruses (HPVs) frequently cause cancer. Yet these SCCs are often HPV negative. FA patients have a genetic defect in their capacity to repair damaged DNA. HPV oncogenes cause an accumulation of DNA damage. We hypothesize, therefore, that DNA damage induced by HPV leads to an accumulation of mutations in patients with FA deficiency and that such mutations allow HPV-driven cancers to become independent of the viral oncogenes. Consistent with this hypothesis, we found that cervical cancers arising in HPV16 transgenic mice with FA deficiency frequently escape from dependency on the HPV16 oncogene that drove its development. Our report provides further support for vaccination of FA patients against HPVs and argues for the need to define mutational profiles of SCCs arising in FA patients in order to inform precision medicine-based approaches to treating these patients.

## Importance

Fanconi anemia (FA) patients are at high risk for developing squamous cell carcinoma (SCC) at sites where high-risk human papillomaviruses (HPVs) frequently cause cancer. Yet these SCCs are often HPV negative. FA patients have a genetic defect in their capacity to repair damaged DNA. HPV oncogenes cause an accumulation of DNA damage. We hypothesize, therefore, that DNA damage induced by HPV leads to an accumulation of mutations in patients with FA deficiency and that such mutations allow HPV-driven cancers to become independent of the viral oncogenes. Consistent with this hypothesis, we found that cervical cancers arising in HPV16 transgenic mice with FA deficiency frequently escape from dependency on the HPV16 oncogene that drove its development. Our report provides further support for vaccination of FA patients against HPVs and argues for the need to define mutational profiles of SCCs arising in FA patients in order to inform precision medicine-based approaches to treating these patients.

## INTRODUCTION

Fanconi anemia (FA) is a rare, recessive, autosomal disease associated with bone marrow failure, acute myelogenous leukemia (AML), and squamous cell carcinoma (SCC), the latter arising at sites known to be associated with HPV-driven cancers, specifically, the female reproductive tract and the head/neck region (1). Cells from FA patients are hypersensitive to DNA interstrand-cross-linking agents and have chromosomal abnormalities (2–4). So far, 16 FA-associated (*fanc*) genes have been identified, all of which are involved in the repair of DNA damage. These genes encode proteins that assemble at sites of damaged DNA at stalled replication forks (5). This leads to the monoubiquitination of the “ID” complex composed of FANCI and D2 proteins (6). This monoubiquitination step leads to the recruitment of effector proteins FANCD1, J, N, O, and P, which contribute to the repair of the damaged DNA. Disruption of the FA pathway leads to genomic instability, and this is thought to put FA patients at increased risk of developing cancer.

Human papillomaviruses (HPV) are small double-stranded DNA viruses that infect stratified epithelium and cause warts and cancers in the anogenital track and head and neck region. FA patients have a high frequency of infections by HPVs (7). Almost all cases of cervical cancer (8) and the majority of other anogenital cancers and 25% of head and neck cancers (9) are etiologically associated with a subset of mucosotropic HPVs, particularly the HPV16 genotype. HPV16 encodes three oncogenes, E5, E6, and E7. Among the genes encoding these 3 oncoproteins, E7, best known for its ability to inactivate the cellular tumor suppressor pRb, is the most potent oncogene (10–12). We have shown previously that *K14E7* transgenic mice, which constitutively express the HPV16 E7 oncogene in stratified squamous epithelium, develop cervical cancer when treated with estrogen (11, 13). Prior studies have demonstrated that multiple human cervical cancer-derived cell lines are dependent upon continued expression of the viral E6 and E7 oncogenes for their continued growth and transformed properties (14–17). Likewise, we have shown, using *Bi-L E7* transgenic mice in which the HPV16 E7 is under the control of a tetracycline-regulated transcription factor, that cervical cancers require the continued expression of HPV16 E7 in FA pathway-sufficient mice (18). This dependency on E7 was observed even in the context of constitutive expression of HPV16 E6, the second viral oncogene expressed in HPV-associated cancers (19). Thus, HPV16-associated cancers on the FA pathway-sufficient background are addicted to the E7 oncogene.

Because FA patients develop cancers in the (female) anogenital tract and in the oral cavity, sites at which HPVs are known to cause cancer, the relationship between FA pathway deficiency and HPV infection has been of intense interest. *In vitro* studies demonstrated that HPV16 E7 accelerated genomic instability when the FA pathway was compromised (20), and in HPV16-positive human keratinocytes, deficiency in the FA pathway significantly increased epithelial hyperplasia and the productive stage of the HPV life cycle (21, 22). *In vivo* studies have demonstrated that HPV16 transgenic mice that are deficient for the FA pathway are at increased risk of developing cervical cancer (23) as well as head and neck cancer (24). So how do we account for the fact that the squamous cell carcinomas arising in FA patients in the anogenital tract and the head and neck region are largely HPV negative?

We hypothesized that in FA patients, who are commonly infected with HPVs and who frequently develop squamous cell carcinomas of the (female) reproductive tract and the head/neck region, the dependency upon continued expression of the HPV oncogenes is lost because the absence of a functional FA pathway leads to the accumulation of mutations, including ones that make the cancers no longer dependent upon the HPV oncogenes. To test this hypothesis, we used our animal model for HPV16-associated cancers of the female reproductive tract. Specifically, we put *Bi-L E7*/*K14-tTA* mice, in which we can temporally regulate expression of the dominant HPV16 oncogene, on either the *FancD2*-sufficient background or the *FancD2*-deficient background and chronically treated the mice with estrogen to induce cervical cancer. We observed that E7 induced cervical cancers and that *FancD2* deficiency increased the incidence of cancer. When E7 expression was repressed on the *FancD2*-sufficient background, cancers and high-grade dysplasia regressed, consistent with our earlier studies (18). However, on the FA pathway-deficient background, cancers and precancerous lesions remained even though E7 was no longer expressed. This result indicates that cervical cancers do not require the continuous expression of E7 on the FA pathway-deficient background. These findings may reconcile the paradoxical findings that, while FA patients are highly susceptible to HPV infection and develop cancers at sites known to be associated with HPVs, these cancers are not found to be positive for HPVs.

## RESULTS

### The incidence of HPV-associated cancers was increased in *FancD2*-deficient mice.

To examine if HPV16 E7 increases the incidence of cervical cancer, *Bi-L E7* and *K14-tTA* transgenic mice were crossed to *FancD2* heterozygous mice to generate *Bi-L E7*/*FancD2*^+/*−*^ and *Bi-L E7*/*FancD2*^+/*−*^ mice. These mice were then crossed to each other to create *FancD2*^+/+^, *Bi-L E7*/K14–*tTA*/*FancD2*^+/+^, *FancD2*^−/*−*^, and *Bi-L E7*/*K14-tTA*/*FancD2*^−/*−*^ mice. To induce cervical cancer, mice were treated with 17β-estradiol, which synergizes with HPV oncogenes, for 10 months. At the end of 10 months, we sacrificed the mice, harvested their reproductive tracts, fixed the samples in 4% paraformaldehyde, embedded the samples in paraffin, and sectioned the tissues. We stained tissue sections with hematoxylin and eosin (H&E) and scored every 10th section histopathologically for the worst grade of disease, classifying the tissues as representing hyperplasia, low-grade dysplasia (cervical intraepithelial neoplasia 1 [CIN I]), midgrade dysplasia (CIN II), high-grade dysplasia (CIN III), or cancer.

As summarized in Table 1, no cervical cancers were observed in *FancD2*^+/+^ and *FancD2*^−/*−*^ mice, indicating that *FancD2* deficiency alone is not sufficient to cause cervical cancer, supporting our previous observations (23). Nine percent of the *Bi-L E7*/*K14*–*tTA*/*FancD2*^+/+^ mice developed cancer. The severity of disease (this assesses all stages of disease from hyperplasia to cancer) in the *Bi-L E7*/*K14*–*tTA*/*FancD2*^+/+^ mice was significantly worse than in the *FancD2*^+/+^ mice (*P* = 0.008). In contrast, 56% of the *Bi-L E7*/*K14-tTA*/*FancD2*^−/*−*^ mice developed cancer. Again, their disease was significantly more severe than that in the *FancD2*^−/*−*^ mice (*P* = 0.013). The cancer incidence in *Bi-L E7*/*K14-tTA*/*FancD2*^−/*−*^ mice was significantly higher than in the *Bi-L E7*/K14–*tTA*/*FancD2*^+/+^ mice (p = 0.009), indicating that FA deficiency facilitates development of cervical cancer.

**TABLE 1:** Incidence of cervical disease in mice

Genotype (*n*^a^)	Dox treatment	Grade of cervical disease				
		Hyperplasia	CIN I	CIN II	CIN III	Cancer (%)
*FancD2*^+/+^ (7)			3	4		
*Bi-L E7*/*K14*–*tTA*/*FancD2*^+/+^ (23)^b^			2	10	9	2 (9)
*Bi-L E7*/*K14*–*tTA*/*FancD2*^+/+^ (19)^b^	1 mo	1	12	6		
*FancD2*^−/*−*^ (6)		1	2	2		
*Bi-L E7*/*K14-tTA*/*FancD2*^−/*−*^ (16)^c^			3	3	1	9 (56)^d^
*Bi-L E7*/*K14-tTA*/*FancD2*^−/*−*^ (15)^c^	1 mo	2	5	3		5 (33)^d^

a *n*, total number of mice examined for each genotype.

b *P* = 2.644 × 10^−6^ (comparing the average severity of disease of *Bi-L E7*/*K14tTA*/*FancD2*^+/+^ to that of *Bi-L E7*/*K14-tTA*/*FancD2*^+/+^ [Dox] using a two-sided Wilcoxon rank sum test). *P* = 7.9 × 10^−6^ if we do the same comparison but exclude cancers.

c *P* = 0.085 (comparing the average severity of disease of *Bi-L E7*/*K14tTA*/*FancD2*^−/*−*^ to that of *Bi-L E7*/*K14-tTA*/*FancD2*^−/*−*^ using a two-sided Wilcoxon rank-sum test). *P* = 0.0158 if we do the same comparison but exclude cancers.

d *P* = 0.2852 (comparing the incidence of cancer of *Bi-L E7*/*K14tTA*/*FancD2*^+/+^ to that of *Bi-L E7*/*K14-tTA*/*FancD2*^+/+^ [Dox] using a two-sided Fisher exact test).

### Severity of disease and cancer incidence in the cervix are independent of continued expression of HPV16 E7 on the *FancD2*-deficient background but not on the *FancD2*-sufficient background.

**FIG 1:**
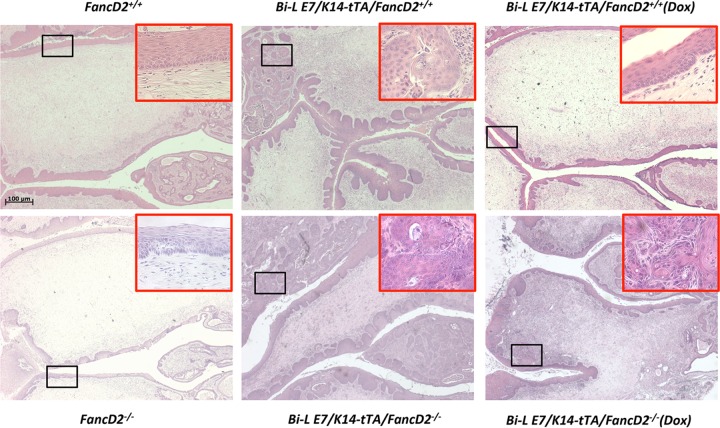
Histopathology of representative female reproductive tracts. Low-magnification images of the cervices stained with hematoxylin and eosin are shown. A high-magnification image of cancers (*Bi-L E7*/*K14*–*tTA*/*FancD2*^+/+^, *Bi-L E7*/*K14-tTA*/*FancD2*^−/*−*^, and *Bi-L E7*/*K14-tTA*/*FancD2*^−/*−*^ [Dox]) or cervical epithelium (*FancD2*^+/+^, *FancD2*^−/*−*^, and *Bi-L E7*/*K14*–*tTA*/*FancD2*^+/+^ [Dox]) is shown in the upper right corner of each image. Black boxes indicate the approximate locations of the enlarged areas.

We have previously shown that, on the *FancD2*-sufficient background, cervical cancers are addicted to E7; turning off expression of E7 in the context of *Bi-L E7*/*K5-tTA* mice by administering doxycycline (Dox) led to regression of cervical neoplasia (18). We found the same to be true in our hands in the context of *Bi-L E7*/*K14*–*tTA*/*FancD2*^+/+^ mice, upon administration of doxycycline for the last month before the endpoint (Table 1). The decrease in cancer incidence and in the severity of disease was highly significant. Examples of histology results are shown in Fig. 1. To examine if cervical cancers arising in *Bi-L E7*/*K14-tTA* mice on the *FancD2*-deficient background remain dependent upon continued expression of E7, we likewise administered doxycycline to *Bi-L E7*/*K14-tTA*/*FancD2*^−/*−*^ mice for the last month on estrogen to repress E7 expression. On the FA pathway-deficient background, cancers still remained even though E7 was no longer expressed, indicating that these cancers had become independent of E7. However, the overall severity of cervical disease, representing scores for all stages of neoplastic disease, was decreased though this decrease did not reach the point of being statistically significant. This difference between retention of cancers versus a marginal decrease in the severity of disease may suggest that precancerous lesions have not yet become completely independent of E7.

### **MCM7 expression patterns confirm that cancers retained in *Bi-L E7***/***K14-tTA***/***FancD2***^−/***−***^** mice treated with doxycycline are not dependent upon continued expression of HPV16 E7.**

**FIG 2:**
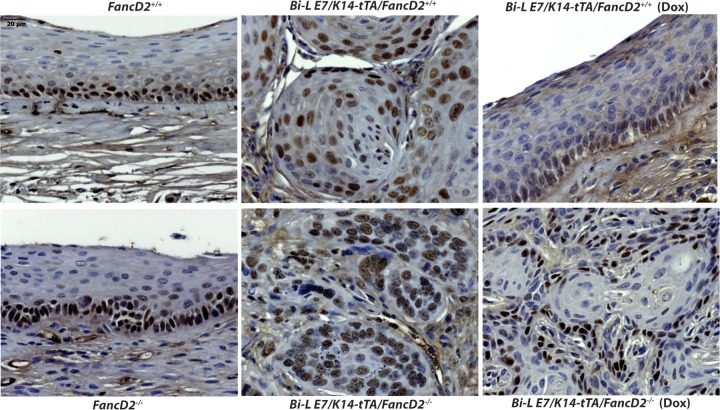
Expression of MCM7, an E7-specific biomarker in cervical epithelium and cancers; evidence for temporal regulation of E7 expression. Representative immunohistochemistry images are shown at high magnification. The images for *FancD2*^+/+^, *Bi-L E7*/K14–*tTA*/*FancD2*^+/+^ (Dox), and *FancD2*^−/*−*^ are showing endocervical epithelium since they did not develop any cancer. The images for *Bi-L E7*/*K14*–*tTA*/*FancD2*^+/+^, *Bi-L E7*/*K14-tTA*/*FancD2*^−/*−*^, and *Bi-L E7*/*K14-tTA*/*FancD2*^−/*−*^ (Dox) are showing cervical cancers. Brown nuclei represent MCM7-positive cells, and hematoxylin (blue) was used to counterstain nuclei. Scale bar, 20 µm.

The gene encoding mouse anti-minichromosome maintenance protein 7 (MCM7) is an E2F-responsive gene which is negatively regulated by pRb and is used as a surrogate for E7 expression/function in humans and mice (13, 25, 26). MCM7 is normally expressed in the basal cells rather than in the suprabasal cells in our mouse models (25). To determine whether the expression pattern of MCM7 is changed in mice when E7 is repressed, we performed immunohistochemistry on sections of female reproductive tracts (Fig. 2). In *Bi-L E7*/*K14*–*tTA*/*FancD2*^+/+^ mice, MCM7 expression was upregulated in cancers and epithelium as evidenced by expression of MCM7 throughout the epithelial cells within these tissues, but when E7 expression was repressed by doxycycline, MCM7 was limited in its expression in the poorly differentiated cells within the epithelium (no cancers were present in this population of mice, which is reflective of their continued dependence on E7—see Table 1). In *Bi-L E7*/*K14-tTA*/*FancD2*^−/*−*^ mice, we likewise observed expression of MCM7 throughout the epithelial cells within the cancers and the cervical epithelium. However, in *Bi-L E7*/*K14-tTA*/*FancD2*^−/−^ mice treated with doxycycline, expression of MCM7 was restricted in its expression to the poorly differentiated cells not only within the epithelium but also in the cancers, confirming that E7 is no longer expressed in those cancers. These findings indicate that the cancers retained in *Bi-L E7*/*K14-tTA*/*FancD2*^−/*−*^ mice treated with doxycycline do not depend on E7 activity. Our results confirm that the cancers persisting in the *Bi-L E7*/*K14-tTA*/*FancD2*^−/*−*^ mice treated with doxycycline no longer depend upon the continued expression of HPV16 E7.

### **Proliferation is upregulated in E7-expressing mice and in *bi-l e7***/***k14-tta***/***FancD2***^−/***−***^** mice treated with doxycycline.**

**FIG 3:**
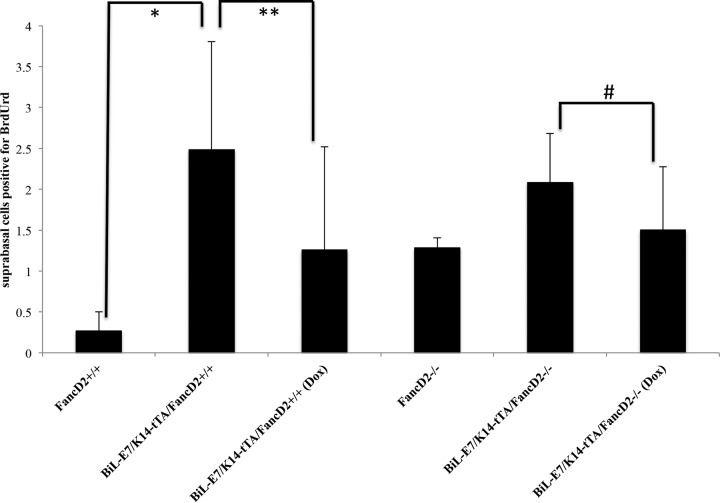
Cellular proliferation levels in cervical epithelium. To monitor newly synthesized DNA, the cervical epithelia were stained with BrdUrd (a complete description of the protocol used is provided in Materials and Methods). For each genotype, at least 3 mice were randomly selected and eight image frames of cells at the cervical epithelia (non-tumor-bearing regions) were quantified. The numbers of BrdUrd-positive cells and the total numbers of cells were plotted. Using a two-sided Wilcoxon rank sum test, statistical analyses were performed. *, *FancD2*^+/+^ versus *Bi-L E7*/*K14*–*tTA*/*FancD2* (*P* = 0.03571). **, *Bi-L E7*/*K14*–*tTA*/*FancD2*^+/+^ versus *Bi-L E7*/*K14*–*tTA*/*FancD2*^+/+^ (Dox) (*P* = 0.0556). #, *Bi-L E7*/*K14-tTA*/*FancD2*^−/*−*^ versus *Bi-L E7*/*K14-tTA*/*FancD2*^−/*−*^ (Dox) (*P* = 0.6286). For *FancD2*^+/+^ versus *FancD2*^−/*−*^, *P* = 0.5379.

We were interested in knowing whether cell proliferation is affected by the presence or absence of E7 and the FA pathway. To study this, mice were injected with the nucleotide analog 5-bromo-2′-deoyuridine (BrdUrd) 1 h before sacrifice. We carried out immunohistochemical staining for BrdUrd to measure newly synthesized DNA, and BrdUrd-positive cells in the suprabasal layer were counted and quantified as indicated in Fig. 3. *Bi-L E7*/*K14*–*tTA*/*FancD2*^+/+^ mice showed increased cell proliferation compared to *FancD2*^+/+^ mice. We observed a decrease in proliferation in *Bi-L E7*/*K14*–*tTA*/*FancD2*^+/+^ mice treated with doxycycline that was marginally significant (*P* = 0.056) compared to that observed in *Bi-L E7*/*K14*–*tTA*/*FancD2*^+/+^ mice not treated with doxycycline. However, in *FancD2*-deficient mice, turning off expression of E7 did not lead to a decrease in cell proliferation (*P* = 0.6286). Comparing cervical tissues from *FancD2*^+/+^ and *FancD2*^−/*−*^ mice, we saw a trend toward higher proliferation rates in the *FancD2*^−/*−*^ group, consistent to what we observed in our prior study (23). These results indicate that *FancD2* deficiency substitutes for the need of E7 to induce cell proliferation.

## DISCUSSION

### HPV-associated cancers become independent of HPV16 oncogene E7 in the absence of a functional FA pathway.

In the current study, we addressed whether HPV16 E7 is required to maintain HPV-associated cancers when the FA pathway is disrupted. To investigate this issue, we utilized Tet-regulatable *Bi-L E7*/*K14-tTA* transgenic mice to control E7 expression via administration of the tetracycline analog doxycycline (19). Consistent with our previous studies (18, 19), E7 was required for cervical cancers in the presence of a functional FA pathway. However, we observed that E7-driven cancers in *FancD2*-deficient mice become independent of continued expression of E7. Turning off expression of E7 led to only marginal regression of overall disease severity in the study of the lower reproductive tract (for *Bi-L E7*/*K14-tTA*/*FancD2*^−/*−*^ versus *Bi-L E7*/*K14-tTA*/*FancD2*^−/*−*^ [Dox], *P* = 0.08; *P* = 0.16 if we exclude cancers). In contrast, the difference in the overall severities of disease when E7 was turned off in the *FancD2*-sufficient background was highly significant (for *Bi-L E7*/*K14*–*tTA*/*FancD2*^+/+^ versus *Bi-L E7*/*K14*–*tTA*/*FancD2*^+/+^ [Dox], *P* = 2.644 × 10^−6^; *P* = 7.9 × 10^−6^ if we exclude cancers). This indicates that not only the cancers but even some precancerous lesions must escape from their dependency on E7 in the *FancD2*-deficient mice.

We also observed that FA deficiency marginally increased the disease severity (for *Bi-L E7*/*K14*–*tTA*/*FancD2*^+/+^ versus *Bi-L E7*/*K14-tTA*/*FancD2*^−/*−*^, *P* = 0.11) while greatly increasing the incidence of cancers (for *Bi-L E7*/*K14*–*tTA*/*FancD2*^+/+^ versus *Bi-L E7*/*K14-tTA*/*FancD2*^−/*−*^, *P* = 0.0026). This suggests that E7 expression and FA deficiency synergize to worsen the stage of neoplastic disease in the female reproductive tract, further demonstrating that the FA pathway counteracts the contribution of E7 to carcinogenesis. A simple explanation for this synergy is that E7 induces DNA damage that fails to be repaired on the FA-deficient background (27). What genetic/epigenetic alterations accumulate in the FA-deficient context that render cancers or even precancerous lesions no longer dependent upon HPV oncogenes is an important issue that remains unresolved both in this preclinical animal model setting and in humans. With the development of emerging technologies for deep sequencing of archival, paraformaldehyde-fixed, paraffin-embedded tissues at even the single-cell level, we and others in the FA field hope to be able to resolve this issue in the near future. Such knowledge could lead to identifying pathway-targeted therapies for use in treating cancers arising in FA patients, for whom traditional chemotherapy and radiotherapy cannot be used. It may also help us learn if there are genetic and/or epigenetic signatures left behind by HPV oncogenes that could allow us to identify HPV-negative cancers that initially arose from an HPV infection. Such cancers could occur even in an FA-sufficient context due to the ability of HPV oncogenes to drive mutagenesis through their induction of DNA damage (28, 29), aneuploidy (28), and genome editing (30) and their ability to reprogram the cell epigenetically (31) as well as to influence of the microenvironment such as by the mutagenic effects of reactive oxygen species elicited due to local inflammatory responses. Having a signature for HPV-initiated cancers could allow us to learn if there is a broader role for this highly oncogenic virus than is currently appreciated.

Escape from dependency on an oncogene has been reported in other cancer contexts. Gunther et al. observed that mammary tumors were dependent on the continuous expression of Wnt1 in wild-type mice but that, on the *p53*^+/*−*^ background, repression of Wnt1 did not lead to regression of tumors (32). Similarly, in the study by D’Cruz et al., mammary tumors in mice regressed when c-Myc expression was silenced but tumors bearing the *de novo* mutation of *Ras* did not regress, indicating that these tumors had become independent of c-Myc (33). In these examples, it is likely that the direct effect of the p53 heterozygous state (and/or loss of heterozygosity [LOH] at the p53 allele in the tumors) or the activating mutation in ras contributes to the loss of addiction to Wnt1 or c-Myc, respectively. In our studies, the FA-deficient state potentially indirectly contributed to the loss of addiction to E7 through an accumulation of mutations in cellular genes that then supplanted E7’s oncogenic activities. In this regard, E7 may induce DNA damage at least in part through its inactivation of the tumor suppressor pRb and of related pocket proteins, p107 and/or p130 (23). Importantly, this induction of DNA damage by E7 is accentuated in the FA-deficient context both *in vitro* (20) and *in vivo* (27). These observations may provide a mechanistic underpinning to our observation that whereas *FancD2*^−/*−*^ mice that are not transgenic for HPV16 E7 are not more susceptible to cervical carcinogenesis than *FancD2*^+/+^ mice, the *FancD2*^−/*−*^ mice that are transgenic for HPV16 E7 are more susceptible to cervical carcinogenesis than their *FancD2*^+/+^ counterparts. That is, there is a synergy between E7 and FA deficiency in causing cervical cancer.

### The relationship between HPV and Fanconi anemia: implications for the clinical treatment of FA patients.

FA patients are at a high risk of being infected with HPVs and developing cancers known to be associated with HPVs in the general population. However, the association of HPVs with SCCs in FA patients has been highly debated. Whereas Kutler et al. (34) reported that 84% of head and neck cancers were HPV positive, van Zeeburg et al. (35) observed that 0 of 16 head and neck cancers were HPV positive. Recently, Alter et al. (36) detected only 1 HPV16-positive vulvar squamous cell carcinoma among 4 anogenital cancers and 0 of 4 head and cancers from FA patients. Those studies used different HPV detection methods and were performed on samples from patients living in different geographical areas of the world; however, the data as a whole would suggest that for many FA patients, the SCCs that they develop are HPV negative.

Our current studies could provide an explanation for why FA patients, while frequently infected with HPVs and frequently developing cancers at sites known to be associated with HPV-induced carcinogenesis, often do not contain HPVs in their cancers arising at such sites. Specifically, we posit that, in FA patients, the paucity of HPV found in anogenital tract and head and neck cancers is a consequence of their cancers becoming independent of a need for continued expression of HPV oncogenes. Our data, however, also show that in the absence of HPV16 E7, FA pathway deficiency is not sufficient to cause these cancers. This observation, which confirms our earlier observations, suggests that HPV may still be the driver of SCCs arising in FA patients, even if the cancers that arise then become independent of the virus. Thus, we think that it remains prudent to vaccinate FA patients as a means of preventing the onset of anogenital tract and head and neck region cancers.

## MATERIALS AND METHODS

### Transgenic mice.

*Bi-L E7* (18), *K14-tTA* (37), and *FancD2*^−/*−*^ (38) transgenic mice have been described previously. One hour before sacrifice, mice were injected with 0.3 ml 5-bromo-2′-deoyuridine (BrdUrd; 12.5 mg/ml). Female lower reproductive tracts were harvested, fixed for 24 h at 4°C in 4% paraformaldehyde, embedded in paraffin, sectioned, and stained with H&E for histologic analysis of neoplastic and dysplastic disease.

### Treatment with doxycycline for repression of expression of E7 in mice.

Doxycycline-containing chow (Bio-Serv) (2 g/kg of body weight) was used to repress the expression of the *Bi-L E7* transgene at the indicated time points.

### Immunohistochemistry and immunofluorescence.

Immunohistochemistry and immunofluorescence procedures were performed as described previously (25). The following primary antibodies were used: mouse anti-BrdUrd (Calbiochem Immunochemicals) (1:50) and mouse anti-minichromosome maintenance protein 7 (MCM7; NeoMarkers Corp.) (1:200).

### Quantification of BrdUrd-positive cells.

Sections from at least 3 mice from each genotype were scored for positivity for BrdUrd (using at least 8 frames/sample at ×20 magnification).

### Statistical analysis.

The MSTAT software program (http://mcardle.wisc.edu/mstat/) was used for determining statistical significance in this study. The Wilcoxon rank sum test was used to determine the average severity of disease and the number of BrdUrd-positive cells in comparisons between groups of mice. The Fisher exact test was used to determine the significance of cancer incidence in comparisons between groups of mice.
